# The effect of military clothing on gunshot wound patterns in a cadaveric animal limb model

**DOI:** 10.1007/s00414-019-02135-9

**Published:** 2019-08-14

**Authors:** Tom Stevenson, Debra J. Carr, Iain E. Gibb, Sarah A. Stapley

**Affiliations:** 1 Impact and Armour Group, Centre for Defence Engineering, Cranfield University, Defence Academy of the United Kingdom, Shrivenham, SN6 8LA UK; 2 Cranfield Forensic Institute, Cranfield University, Defence Academy of the United Kingdom, Shrivenham, SN6 8LA UK; 3 Present Address: Defence and Security Accelerator, Cranfield University, Porton Down, Salisbury, Wiltshire SP4 0JQ UK; 4 Centre for Defence Radiology, at c/o Sickbay, HMS Nelson, HMNB Portsmouth, Hampshire, PO1 3HH UK; 5 Royal Centre for Defence Medicine, ICT Building, Research Park, St. Vincent Drive, Birmingham, B15 2SQ UK

**Keywords:** Ballistic, Extremity, Injury, AK47, AK74

## Abstract

The majority of injuries in survivors of gunshot wounds (GSW) are typically to the extremities. Novel wound ballistic research is encouraged to try and capture corporate knowledge on the management of these injuries gained during recent conflicts and understand the wounding patterns seen. With recent work examining the effect of UK military clothing on extremity GSW patterns in a synthetic model, a model with greater biofidelity is needed for ballistic testing. The aim of this study was to assess the effect of UK military clothing on GSW patterns within a cadaveric animal limb model using two types of ammunition commonly used in recent conflicts—7.62 × 39 mm and 5.45 × 39 mm. In total, 24 fallow deer hind limbs were shot, 12 by 7.62 mm projectiles and the remaining 12 shot by 5.45 mm projectiles, further divided into four with no clothing layers (*C*_nil_), four with a single clothing layer (*C*_min_) and four with maximum clothing layers (*C*_max_) as worn on active duty by UK military personnel. Limbs were analysed after ballistic impact using contrast CT scanning to obtain measurements of permanent cavity damage, and results were compared using analysis of variance (ANOVA). Results showed significantly different damage measurements within limbs with *C*_max_ for both ammunition types compared with the other clothing states. This may result in GSWs that require more extensive surgical management, and invites further study.

## Introduction

Whilst fragmentation injuries typically dominate the amount of combat trauma seen in war, gunshot wounds (GSW) are still responsible for extensive numbers within military casualty statistics throughout numerous major conflicts [1–8]. Most recently during the Iraq and Afghanistan conflicts between 2003 and 2014, 24% of all UK military trauma casualties were due to gunshot, making it the second most common mechanism of injury after blast, with 69% of those GSW survivors suffering extremity wounding [8]. Management of these cases have seen the rapid evolution of clinical practice to try and mitigate the complex nature of these injuries [9, 10]. Novel research into wound ballistics is therefore paramount to continue to try and improve overall patient outcomes and to maintain corporate knowledge. Experimental models for such research come in a variety of forms, such as cadaveric human or animal, live animal or synthetic mediums such as soap or gelatine; many of which have been the recent subject of review [11].

The use of gelatine is a relatively cheap and reliable method to investigate wound ballistics. Ten percent by mass gelatine has been validated against live swine thigh muscle tissue, and previous research into mapping wounding patterns from various ammunition types has been conducted [12–15]. However, the use of a homogenously dense material in this way does not offer sufficient biofidelity with respect to the anatomy found within human and animal subjects, i.e. bone, neurovascular structures, skeletal muscle, muscle fascia, subcutaneous fat and skin [11]. As such the use of human or animal tissue is sometimes required to understand the complex interactions faced with a projectile when it enters the anatomy [16–20].

When examining the effect of clothing within these models, there is literature which reports on contamination of wounds (e.g. [19, 21–24], though there are only a small number of studies which investigate the effect of clothing on the wounding pattern itself (e.g. [25–29]).

The aim of this study was to test the effect of UK military clothing on GSW patterns using a cadaveric animal limb model.

## Materials and methods

Ethical approval for this work was granted through the Cranfield University Research Ethics System (CURES/3579/2017).

### Materials

Previous work by this research group has tested the effect of UK military clothing on a 10% by mass gelatine model using quarantined ammunition to represent the typical threat faced by UK service personnel within recent conflicts [6, 29, 30]. For the purposes of the current work, the same quarantined ammunition batches were used.^1^^,^^2^

**Fig. 1:**
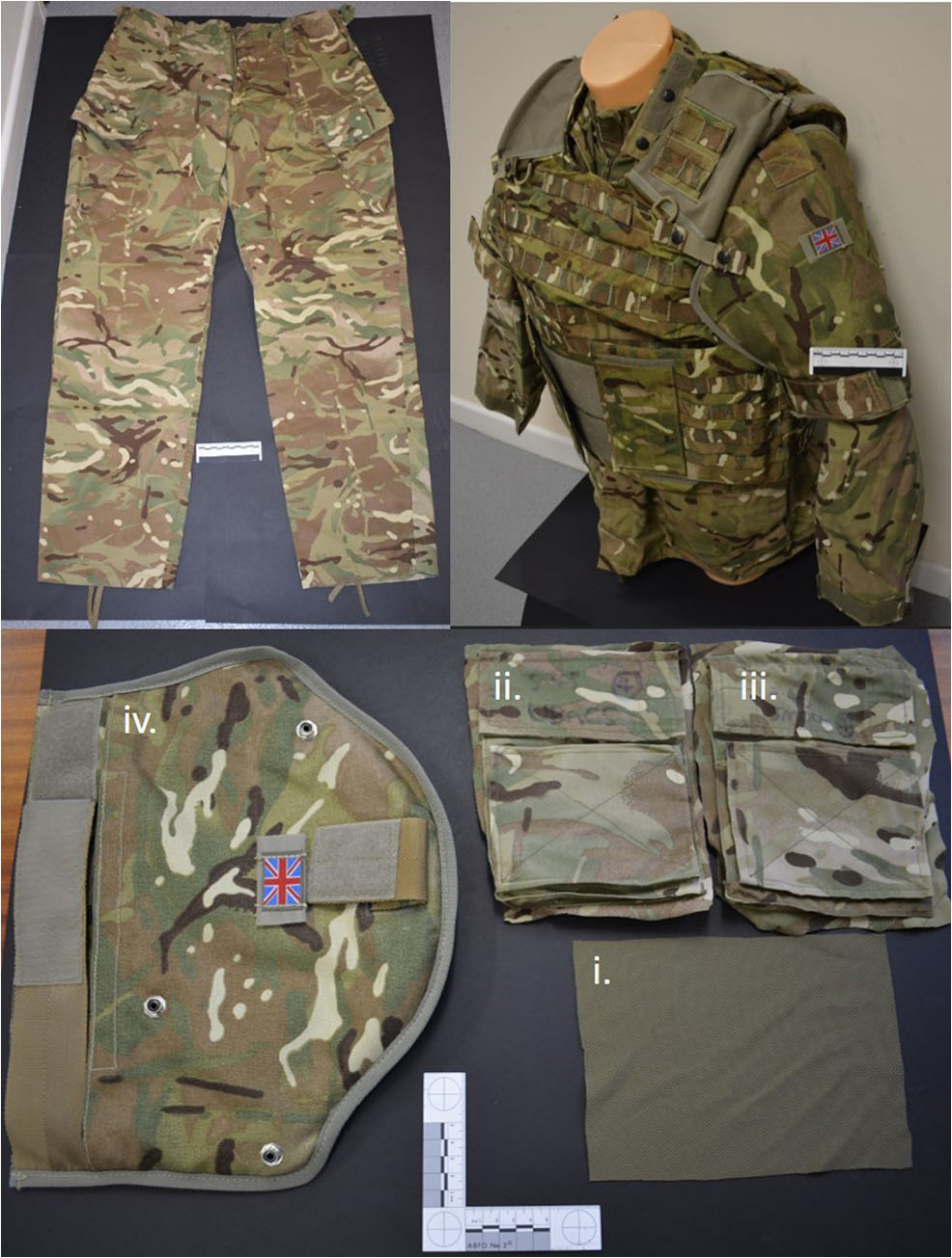
Examples of MTP clothing used—clockwise from top left: MTP trousers; top right: t-shirt, UBACS, smock and brassard as worn by service personnel; bottom: i. t-shirt, ii. UBACS, iii. smock and iv. brassard layers prepared for testing. Laundering detail and fabric analysis data for this clothing used within these experiments is detailed in previously published work [29]

The same standard issue Multi-Terrain Pattern (MTP) UK military clothing states were chosen: a nil clothing state, i.e. no clothes (*C*_nil_), a minimal clothing state, i.e. a single clothing layer taken from MTP trousers (*C*_min_) or a maximum clothing state (*C*_max_), i.e. clothing layers taken from a t-shirt, Under Body Armour Combat Shirt (UBACS), smock and upper arm brassard as worn by UK service personnel (Fig. 1) [29].

Animal tissues selected for testing were fallow deer (*Dama dama*) hind limbs. These were ethically sourced and hunted for entry into the human food chain rather than directly for these experiments. Total body mass for fallow deer is typically 46–94 kg for males and 35–56 kg for females [31]. Fallow deer limbs were chosen due to their muscular nature with less subcutaneous fat, making them more biofidelic to compare with a fit young soldier’s limb, rather than porcine tissue which has a thicker layer of subcutaneous tissues [11, 32, 33]. Deer limbs obtained were of a mass between 9.5 and 13 kg and measuring approximately 280 mm × 700 mm × 100 mm (maximum width × height × maximum thickness). Femurs from deer are similar in morphology with human femurs [34], and therefore it was assumed that soft tissue morphology should follow suit.

In order to judge the suitability of fallow deer limbs to be used as a human tissue surrogate representative of UK service personnel, anthropometric data sources were examined. One survey provided data for the UK population aged between 19 and 65 years gave a 50th percentile body mass of 69 kg for men and women (as a combined group) [35]. Anthropometric data for surveyed UK service personnel gave a 50th percentile body mass for males of 81 kg and 67 kg for females (combined mean of 74 kg) [36]. With one human thigh accounting for 14.2% of stature, this would give an approximate typical thigh mass of 10.5 kg [36]. This suggested that fallow deer limb mass was of reasonable comparison with UK service personnel for this study.

**Fig. 2:**
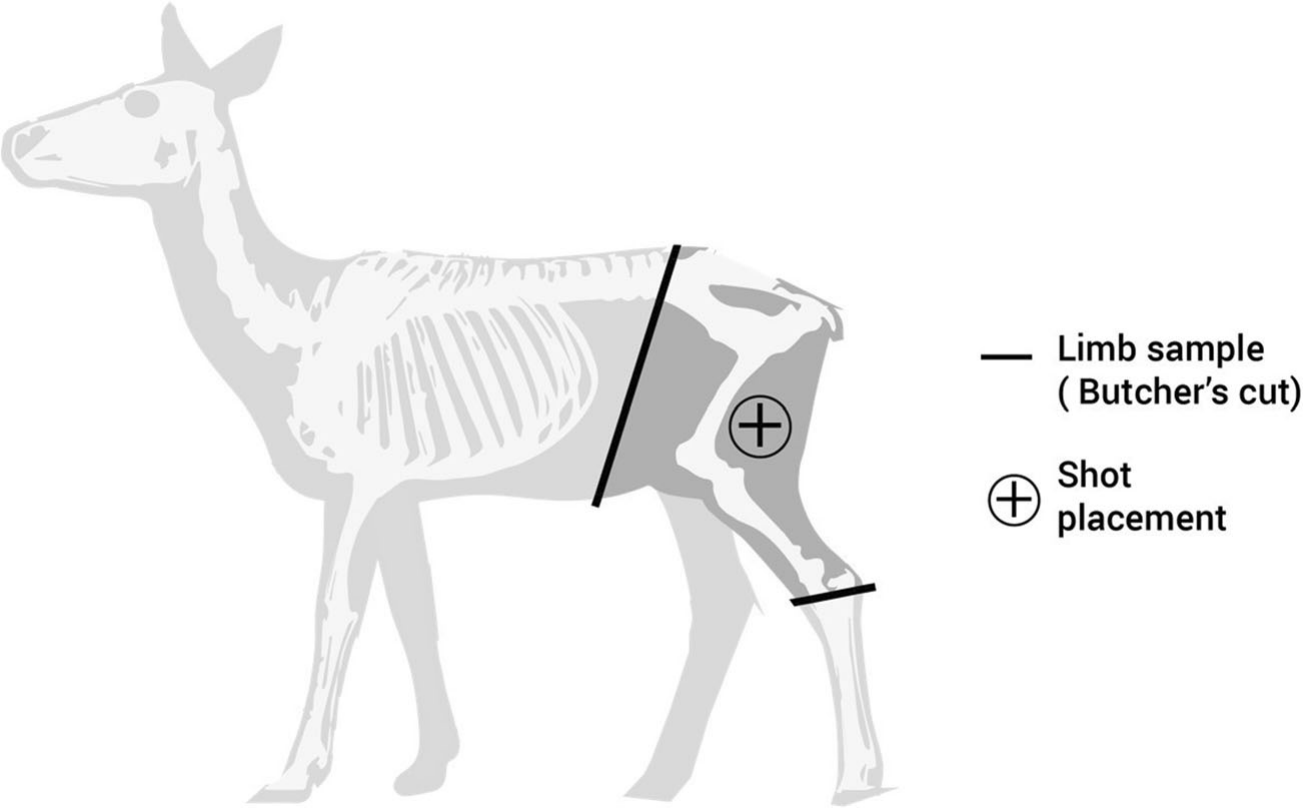
Fallow deer anatomy schematic demonstrating limb preparation and shot placement

**Fig. 3:**
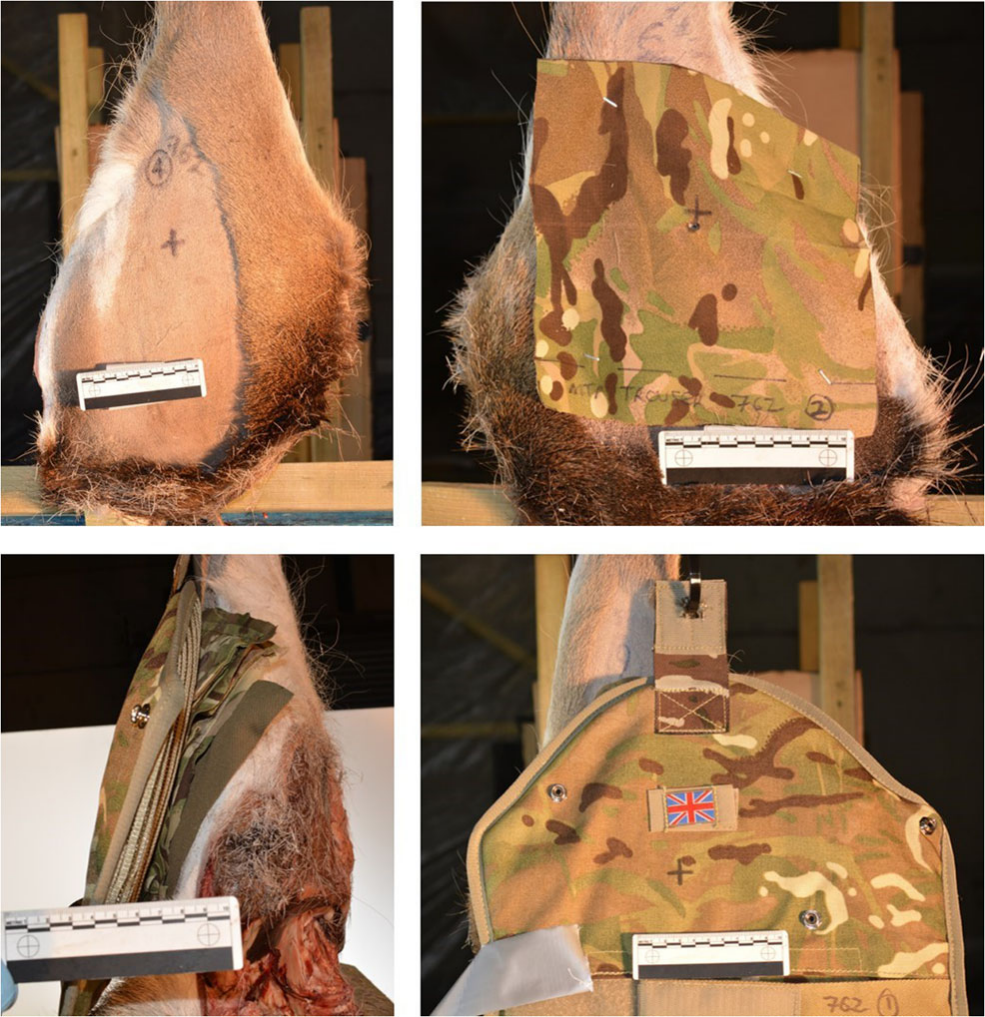
Clockwise from top left: *C*_nil_ front view; *C*_min_ front view; *C*_max_ front view; *C*_max_ side view

The fallow deer limbs were prepared by a professional butcher (Fig. 2) and then shaved (e.g. Figure 3, top left image). Limbs were used both as fresh targets (within 72 h of culling) and also stored by freezing and subsequently defrosted to room temperature over a 72 h period for use, due to differences in availability of range facilities and the acquisition of limbs. The difference in ballistic effects to fresh versus defrosted cadaveric tissue can be considered negligible [37].

## Methods

Fabric samples for *C*_min_ were cut from laundered MTP trousers (250 × 250 mm)^3^ and pinned to the front face of the relevant deer limbs (Fig. 3, top right image). Fabric samples for *C*_max_ were measured and cut in relation to the upper sleeve pocket size on the UBACS and smock (200 × 150 mm)^4^ and placed in layers with the t-shirt layer innermost, then UBACS, smock and finally with the brassard then placed over the top of the other layers (Fig. 1 lower image and Fig. 3 lower images).

Limbs were suspended upside down using an “S”-shaped metal hook looped between the distal tibia and fibula at the ankle joint.

An indoor small arm range was used to fire projectiles from a number 3 proof housing where the end of the barrel was situated at 10 m from the target. Each limb was shot once with the test projectiles. Twelve limbs were shot with 7.62 mm projectiles, and the remaining 12 limbs were shot with 5.45 mm projectiles. Four limbs for each ammunition type had either *C*_nil_, *C*_min_ or *C*_max_ added to the impact surface of the limb.

The impact velocity for each projectile was measured using Doppler radar (Weibel W700). High Speed Video (HSV) taken external to the limbs allowed dynamic recording of the wounding patterns as they occurred, with cameras placed obliquely facing both the entrance^5^ and exit^6^ surfaces of samples. Qualitative examination of GSW patterns was conducted using Phantom Software (Visions Research, Phantom Camera Control Application 2.6).

**Fig. 4:**
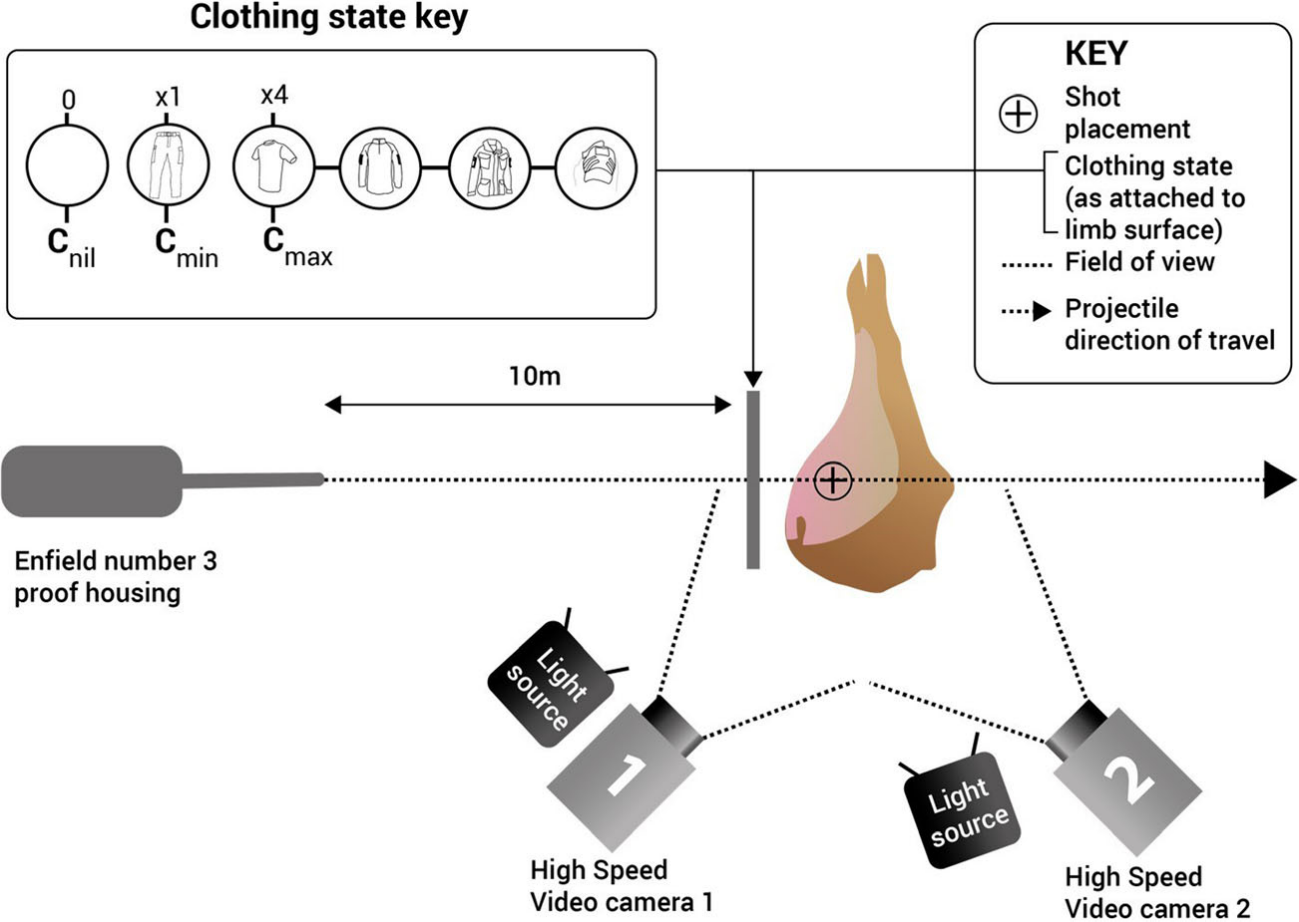
Schematic demonstrating the setup of limbs for shooting with each projectile type

A schematic of the experimental setup is shown in Fig. 4.

**Fig. 5:**
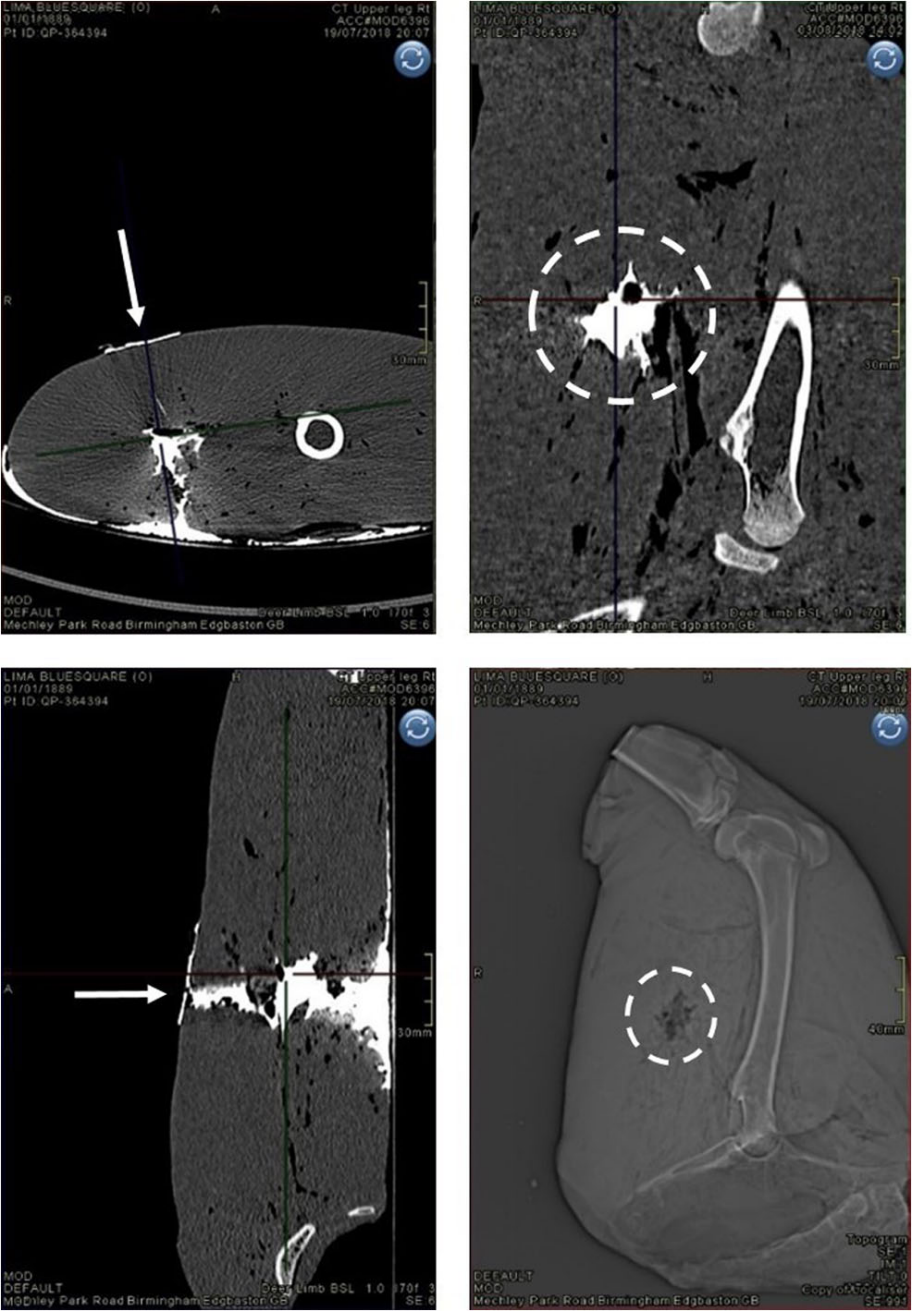
Arrows indicate projectile direction of travel, dotted circles indicate coronal section view of GSW track (in sagittal plane of limb)—clockwise from top left—contrast image, axial plane; contrast image, sagittal plane; X-ray scout view, sagittal plane; contrast image, coronal plane

**Fig. 6:**
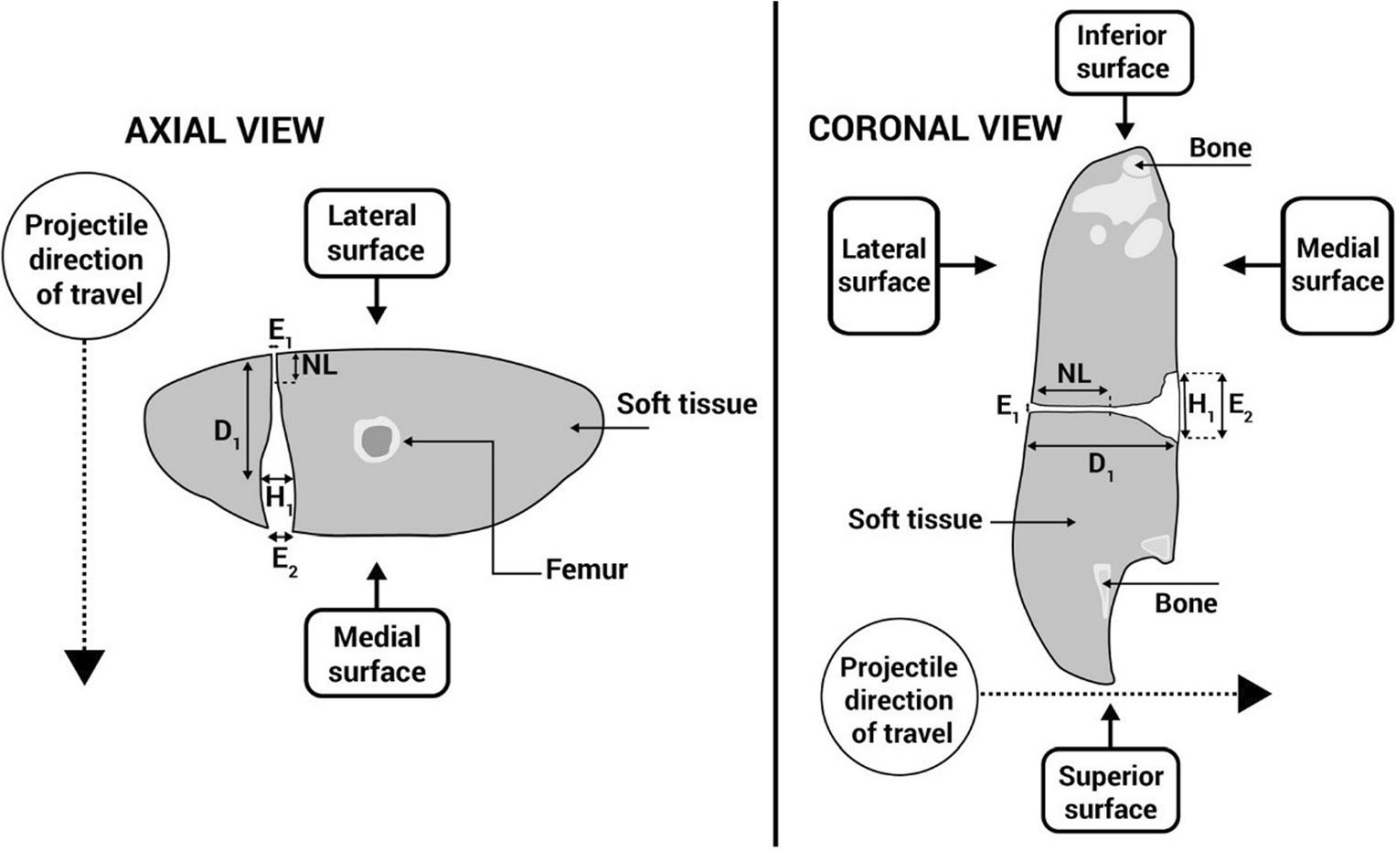
Schematic demonstrating CT scan measurements taken in axial and coronal planes of view (in this example schematic, H1 and E2 in the coronal view were the same; however, this varied amongst specimens)

The damage to the deer limbs was photographed using a Canon D5100 Digital SLR camera (S/N 6773411). Damage to the deer limbs was measured using a dual source (2 × 64 slice) Multi-Slice Computed Tomography (MSCT) scanner,^7^ with contrast solution injected into the limb wounds to aid visualisation [38]. Scans used a standard adult pelvis protocol (exposure figures were 120 kV and 25–32 mAs) with 1.0 mm slice soft tissue and bony reconstructions in the axial, sagittal and coronal planes (where planes of view were defined in relation to the limb). The parameters of damage were measured from multi-planar reconstructed (MPR) images came from axial and coronal viewing planes (Fig. 5), as part of the Syngo CT2012B software package provided with the CT scanner. These parameters were the neck length (NL) of the GSW, maximum size of the permanent cavity (H1), distance to maximum size of the permanent cavity (D1), entrance wound diameter (E1) and exit wound diameter (E2) (Fig. 6). The parameters were chosen in conjunction with other research quantifying damage from GSW [14, 25, 27–29, 39, 40].

The International Business Machine Corporation’s Statistical Package for Social Services version 24 (IBM® SPSS Statistics v24) analysis of variance (ANOVA) was used to determine the effect of the different clothing states, ammunition type and CT plane on NL, H1, D1, E1 and E2. Homogeneity of variance and normality of data were confirmed with a significance level of 0.05 applied. Where an inequality of error variance in ANOVA testing for exit wound (E2) dimensions was found, likely due to the relatively high coefficients of variation (CV) seen, ellipsoid areas (EA) of the exit wounds were calculated from both coronal and axial measurements on CT scan images (Table 2). Similarly, inequality of error variance led to considering ammunition types separately for D1 measurements. Significant differences due to ammunition type and/or clothing condition were identified using Tukey’s honest significant difference (HSD) test. The main effects and significant interactions only are discussed in the “Results” section.

## Results

Mean impact velocity for the 7.62 mm projectiles was 645 m/s (SD = 8 m/s) and for the 5.45 mm projectiles was 907 m/s (SD = 25 m/s), which were as expected for these ammunition types following previous work [29]. Mean mass for unfired 7.62 mm and 5.45 mm projectiles was 7.9 g (SD = 0.06 g) and 3.4 g (0.03 g) respectively. Examination of HSV indicated small entrance wounds and larger exit wounds.

Evidence of bullet wipe and yarn pull-out on the surfaces of the fabric samples was consistent with that described within the literature [33, 41, 42].

The dimensions collected for the damage to limbs caused by projectiles of both ammunition types for all clothing states are summarised in Table 1. The means, standard deviations (SD) and CVs of EA are shown in Table 2.

**Table 1:** Mean, Standard Deviation (SD) and Coefficient of Variation (CV) for dimensions measured

	NL			D1			H1			E1			E2		
Projectile/clothing state	Mean (mm)	SD (mm)	CV (%)	Mean (mm)	SD (mm)	CV (%)	Mean (mm)	SD (mm)	CV (%)	Mean (mm)	SD (mm)	CV (%)	Mean (mm)	SD (mm)	CV (%)
7.62 mm/*C*_nil_	44.0	16.1	36.5	81.6	4.7	5.7	21.3	12.3	57.7	5.4	0.6	11.9	9.8	3.1	31.8
7.62 mm/*C*_min_	31.2	15.8	50.8	50.0	9.1	18.2	14.6	2.1	14.2	4.6	0.7	15.8	10.9	3.3	30.5
7.62 mm/*C*_max_	35.8	10.2	28.4	68.2	24.6	36.1	26.6	13.4	51.0	5.2	1.1	20.8	24.6	25.6	104.3
5.45 mm/*C*_nil_	33.5	21.5	64.1	56.7	13.1	23.2	23.8	3.8	15.9	3.5	0.9	24.9	19.1	7.6	39.6
5.45 mm/*C*_min_	32.2	37.2	115.3	41.4	22.4	54.1	17.0	5.8	34.2	3.0	1.1	36.4	14.7	5.1	34.4
5.45 mm/*C*_max_	37.2	24.3	65.2	80.1	14.5	18.1	31.2	8.4	26.7	4.9	1.3	27.3	22.9	8.6	37.7

**Table 2:** Mean, SD and CV for exit wound ellipsoid areas (EA)

Projectile/clothing state	EA		
	Mean (mm^2^)	SD (mm^2^)	CV (%)
7.62 mm/*C*_nil_	155.7	83.0	53.3
7.62 mm/*C*_min_	182.9	76.9	42.1
7.62 mm/*C*_max_	1143.8	1456.1	127.3
5.45 mm/*C*_nil_	528.3	307.0	58.1
5.45 mm/*C*_min_	308.2	41.2	13.4
5.45 mm/*C*_max_	884.4	567.1	64.1

ANOVA results are given in Table 3; clothing condition data subgroups identified by Tukey’s HSD are also included.

**Table 3:** ANOVA results

Measurement	ANOVA effects (*F* statistic, *p* value)			Data subsets for clothing state (Tukey’s HSD)	
	Clothing state	Ammunition type	Viewing plane	Group 1	Group 2
NL	*F*_2,36_ = 0.38, *p* = NS	*F*_1,36_ = 0.16, *p* = NS	*F*_1,36_ = 1.44, *p* = NS	No subgroups identified	
D1 (5.45 mm)	*F*_1,17_ = 12.47, *p* ≤ 0.01	N/A	*F*_1,17_ = 6.43, *p* ≤ 0.01	*C*_max_	*C*_min_, *C*_nil_
H1	*F*_2,35_ = 8.14, *p* ≤ 0.01	*F*_1,35_ = 1.60, *p* = NS	*F*_1,35_ = 2.14, *p* = NS	*C*_max_, *C*_nil_	*C*_min_, *C*_nil_
E1	*F*_2,36_ = 6.91, *p* ≤ 0.01	*F*_2,36_ = 18.61, *p* ≤ 0.01	*F*_1,36_ = 0.24, *p* = NS	*C*_max_, *C*_nil_	*C*_min_, *C*_nil_
EA	*F*_2,16_ = 3.54, *p* = NS	*F*_1,16_ = 0.10, *p* = NS	N/A	No subgroups identified	

### Clothing state

Clothing state did not affect NL or EA, though significantly affected D1,^8^ H1 and E1 where *C*_max_ measurements were larger than *C*_min_ measurements in all three categories, respectively (Tables 1 and 3). *C*_max_ also resulted in significantly larger D1 measurements than *C*_nil_ (Table 3). The effect of clothing state on D1 when 7.62 mm projectiles were used did not meet the assumptions of ANOVA.

### Ammunition type

Ammunition type did not affect NL, H1 or EA measurements, though significantly affected E1, where samples had larger entrance wounds when shot by 7.62 mm projectiles compared with those shot by 5.45 mm projectiles (Tables 1 and 3). D1 measurements could not be compared between ammunition types where assumptions of ANOVA were not met.

### Viewing plane

Viewing plane had no effect on NL, H1 or E1 measurements for either axial or coronal views on CT scan images apart from for D1 (Table 3).

All projectiles completely perforated respective limb samples, and no projectile fragments were found to have been retained. Whilst not the focus of this study, none of the limb samples received a femur fracture either directly or indirectly.

## Discussion

**Fig. 7:**
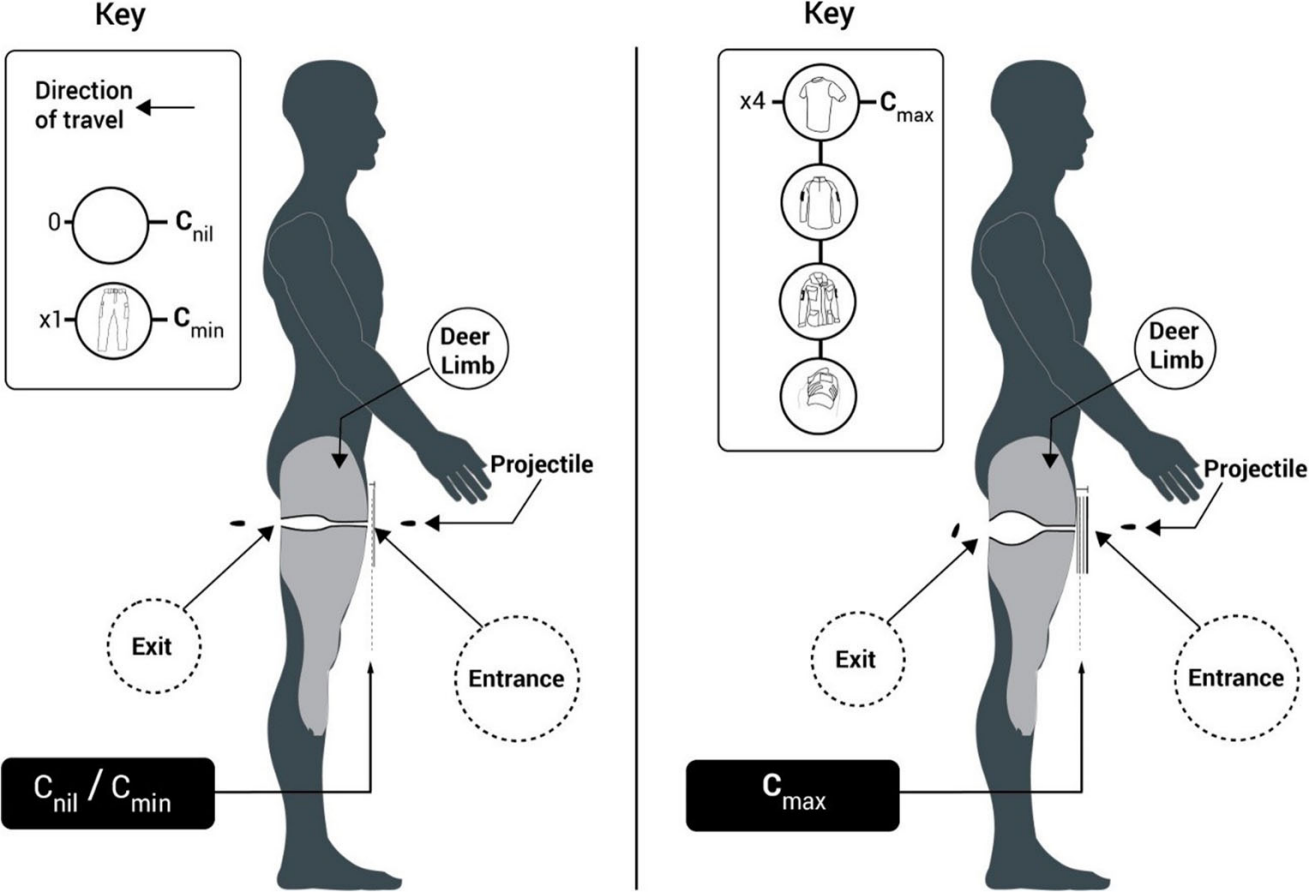
Human anatomical schematic overlaying typical deer limb GSW patterns—*C*_nil_ and *C*_min_ (left) and *C*_max_ (right)

The dimensions of the GSW patterns were the important factor in determining the clinical implications from these results. The importance of neck length on determining the potential level of clinical management required has previously been discussed [29]. Despite the presence of *C*_max_ in the current work not affecting the mean NL for either ammunition type, it did lead to a significantly larger mean H1 for both ammunition types. This suggested that wearing the maximal clothing state could lead to a wound of larger proportions occurring. Translated into a living subject, wounds of a larger proportion imply greater damage has been sustained, or at the very least, more tissue has been involved (Fig. 7). This would necessitate more extensive surgical management such as wound debridement or excision of dead or severely damaged tissue [9, 10]. With a greater amount of tissue loss clinically, either from GSW or from surgery, the resultant effect to the casualty will be increased morbidity with the risk of further procedures and a prolonged recovery or rehabilitation process [6, 8]. The overall finding of worse damage in the presence of *C*_max_ therefore correlates with recent findings on the effect of MTP clothing in a synthetic limb model [29].

Clothing state also affected other elements of the wounding patterns seen across the two ammunition types. The use of 5.45 mm projectiles saw a significant effect with *C*_max_ on mean D1 where distances were found to be longer. The 5.45 mm projectiles were of a lower mass and of a (harder) mild steel core compared with the heavier, lead core 7.62 mm projectiles. This difference in physical properties, in tandem with the respective different velocities of each ammunition type, i.e. 5.45 mm projectiles travelling faster, would suggest that the 5.45 mm projectiles had travelled further before imparting an increased amount of damage when *C*_max_ was present, even though the NL was not statistically different across clothing states. This finding is in part corroborated with previous research demonstrating that 5.45 mm projectiles are more resistant to fragmentation and deformation and leave a more simple wound profile behind [12, 15]. The significantly larger E1 measurement found where *C*_max_ was present was most likely due to increased yaw of projectiles following impact of the clothing layers just prior to impacting the limb surface, leading to a greater cross-sectional surface area of the projectile presenting itself to the tissues. This observation was corroborated with examination of the HSV footage which found that in limbs with *C*_nil_ and *C*_min_ (Fig. 7, left), projectiles exited with minimal, if any, yaw. However, the converse was seen where *C*_max_ was present (Fig. 7, right) with projectiles consistently exiting not only at greatly increased or even maximum yaw but also in some cases violently tumbling end over end. This finding would be in keeping with predictable behaviour of base-heavy projectile types when stability of flight has been lost [33]. Projectile mass also likely influenced the E1 measurements, where entrance wounds were consistently larger in limbs struck by 7.62 mm projectiles for all clothing states.

Viewing planes on CT scan analysis yielded different measurements for each parameter of interest. ANOVA results found a significant difference in such measurements for mean D1 values where 5.45 mm projectiles were used. This finding would be in keeping with asymmetry of wounds sustained within the limbs and the non-uniform shape of wounding patterns often found following GSW [15, 33, 39].

It is crucial to note that with the model considered in this work being cadaveric, there cannot be any comment upon tissue viability following wounding. This therefore requires several assumptions to be made with respect to the wounding patterns seen. It seems reasonable that where the parameters of measurable damage were greater within the permanent cavity, that greater temporary cavitation must have taken place [33]. This, coupled with qualitative analysis of the HSV footage, would suggest that more of the limb tissue was involved with the wounding process [12, 13, 29, 39]. However, it may be that in live tissue, the subsequent recovery of tissue which has been exposed to this level of deformation may be partial or even complete. This notion is observed and well described within one important study by Hopkinson published in 1963 [16]. The study involved using 0.22 in. (5.6 × 35 mm) hornet projectiles to create a soft tissue GSW in live skeletal muscle of sheep limbs and demonstrated that, without any surgical intervention, soft tissue wounds healed well and by three months had replaced all necrotic tissue with new connective tissue and resulted in a negligible amount of fibrotic scar tissue. This would be difficult to prove in human casualties beyond the anecdotal experience of those whom have surgically managed casualties with GSW [6, 10] and warrants further study.

### Limitations

Limitations were the use of one type of clothing (UK military) and two types of military ammunition, each with a single impact velocity. Accepting these limitations was necessary to ensure rigorous variable control could be maintained.

There were several instances where the assumptions of ANOVA were not met, such as with D1 measurements for both ammunition types combined, as well as for samples shot by 7.62 mm projectiles considered separately, and for E2 measurements. These instances were most likely caused by the large variation seen in the data collected, though such variation was accepted under these test conditions.

Other relevant limitations include the tissue selected for study and the dynamic analysis of the wounding patterns. As described in the “Materials” section above, several assumptions had to be made on the use of fallow deer limbs as a tissue surrogate where the authorship was unable to find existing wound ballistic literature using deer limbs for soft tissue GSW modelling. Whilst deer femurs have been shown to be comparable with human femur morphology [34], examination of anthropometric data suggested it could be assumed that the soft tissue morphology would therefore make a reasonable surrogate for ballistic testing [35, 36]. This was therefore a limitation due to there being no open source data available for comparison, though following this work means there is now this available baseline of data from which to build upon. Also, results were only based on post-mortem data, which provided a limitation on interpreting precise clinical application of these results. Finally, the wounding pattern analysis was only on the permanent cavity remaining. As alluded to above, comment on the temporary cavity could only be assumed where the opacity of the deer limbs meant that even with HSV it was not possible to undertake temporary cavity measurements. This was mitigated with knowledge of temporary cavity formation described within existing literature (e.g. [11, 29, 33, 39]).

## Conclusion

*C*_min_ did not affect the damage sustained by a cadaveric deer limb shot by 7.62 mm or 5.45 mm projectiles when compared with a *C*_nil_ clothing state. *C*_max_ significantly affected the damage sustained by a cadaveric deer limb shot by 7.62 mm or 5.45 mm projectiles to both *C*_min_ and *C*_nil_ clothing states. This increases the likelihood of a more complicated surgical intervention being required for human casualties wearing such clothing combinations. Neither iteration of clothing states appeared to affect the propensity of projectile fragmentation or retention nor the risk of femur fracture, though these features were not quantified further.

## Footnotes

1 7.62 × 39 mm wolf hunting cartridges; lead core, 122 grain full metal jacket, lot number F-570, made in Russia, 2006; the core composition was lead mixed with antimony, and jacket composition was steel with internal and external copper wash; mean hardness was 7.8 Hv for the core and 184.4 Hv for the jacket [29].

2 5.45 × 39 mm; mild steel core, 53 grain full metal jacket, lot number 539–04, made in Russia, 2004; with a core composition of steel, a core tip of lead, and a jacket of steel with internal and external copper wash; mean hardness was 814.9 Hv for the core, 3.6 Hv for the core tip, and 188.8 Hv for the jacket [29].

3 C_min_ mean thickness = 0.43 mm; mean mass per unit area = 191.14 g/m^2^ [29]

4 C_max_ mean thickness = 32.26 mm; mean mass per unit area = 7735.17 g/m^2^ [29]

5 Phantom V12 video camera (frames per second = 28,000, shutter speed = 4 μs, resolution = 512 × 384)

6 Phantom V1212 video camera (frames per second = 37,000, shutter speed = 5 μs, resolution = 512 × 384)

7 System SOMATOM Definition AS, 64622, Siemens AG, Wittelsbacherplatz, DE–80333 Munchen, Germany

8 For samples shot by 5.45 mm projectiles only
